# Supplementary material for: Functional insights of novel Bathyarchaeia reveal metabolic versatility in their role in peatlands of the Peruvian Amazon
Source: Microbiol Spectr. 2024 Nov 14;12(12):e00387-24. doi: 10.1128/spectrum.00387-24 (PMC11619403; doi:10.1128/spectrum.00387-24)
Supplement: Supplemental material — Tables S1 to S3; Fig. S1 to S6. [file spectrum.00387-24-s0004.pdf]

Table S1. Study site characteristics of selected peatlands from the Pastaza-Marañón Foreland Basin

	**Buena Vista (BVA)**	**Quistochocha (QUI)**	**Maquía (MAQ)**	**San Jorge** (SJO)
**Longitude (W)**	73°12.146'	73°19.1686'	74°48.492'	73°11.705'
**Latitude (S)**	4°14.378'	3°50.2883'	6°19.381'	4°03.766'
**Peatland Type**	Mixed Forest	Palm Swamp	Open Peatland	Pole Forest
**Depth (cm)**	170	425	430	488
**pH**	5.6 ± 0.1	3.7 ± 0.1	6.1 ± 0.3	2.5 ± 0
**Soil Category**	Minerorophic	Mixed	Mixed	Ombrotrophic

Table S2. Single-copy phylogenetic marker genes used for phylogenic analysis of the Bathyarchaeia MAGs

Pfam	HMM name	Gene Description
PF00121	TIM	Triosephosphate isomerase
PF00164	Ribosom_S12_S23	Ribosomal protein S12/S23
PF00177	Ribosomal_S7	Ribosomal protein S7p/S5e
PF00203	Ribosomal_S19	Ribosomal protein S19
PF00237	Ribosomal_L22	Ribosomal protein L22p/L17e
PF00238	Ribosomal_L14	Ribosomal protein L14p/L23e
PF00252	Ribosomal_L16	Ribosomal protein L16p/L10e
PF00276	Ribosomal_L23	Ribosomal protein L23
PF00297	Ribosomal_L3	Ribosomal protein L3
PF00312	Ribosomal_S15	Ribosomal protein S15
PF00318	Ribosomal_S2	Ribosomal protein S2
PF00344	SecY	SecY translocase
PF00347	Ribosomal_L6	Ribosomal protein L6
PF00366	Ribosomal_S17	Ribosomal protein S17
PF00368	HMG-CoA_red	Hydroxymethylglutaryl-coenzyme A reductase
PF00380	Ribosomal_S9	Ribosomal protein S9/S16
PF00410	Ribosomal_S8	Ribosomal protein S8
PF00411	Ribosomal_S11	Ribosomal protein S11
PF00416	Ribosomal_S13	Ribosomal protein S13/S18
PF00572	Ribosomal_L13	Ribosomal protein L13
PF00573	Ribosomal_L4	Ribosomal protein L4/L1 family
PF00687	Ribosomal_L1	Ribosomal protein L1p/L10e family
PF00709	Adenylsucc_synt	Adenylosuccinate synthetase
PF00749	tRNA-synt_1c	tRNA synthetases class I (E and Q), catalytic domain
PF00750	tRNA-synt_1d	tRNA synthetases class I (R)
PF00827	Ribosomal_L15e	Ribosomal L15
PF00831	Ribosomal_L29	Ribosomal L29 protein
PF01015	Ribosomal_S3Ae	Ribosomal S3Ae family
PF01139	RtcB	tRNA-splicing ligase RtcB
PF01157	Ribosomal_L21e	Ribosomal protein L21e
PF01200	Ribosomal_S28e	Ribosomal protein S28e
PF01201	Ribosomal_S8e	Ribosomal protein S8e
PF01282	Ribosomal_S24e	Ribosomal protein S24e
PF01351	RNase_HII	Ribonuclease HII
PF01655	Ribosomal_L32e	Ribosomal L32e protein family
PF01725	Ham1p_like	Ham1 family

PF01864	CarS-like	CDP-archaeol synthase
PF01866	Diphthamide_syn	Putative diphthamide synthesis protein
PF01948	PyrI	Aspartate carbamoyltransferase regulatory chain
PF01951	Archease	Archease protein family (MTH1598/TM1083)
PF01981	PTH2	Peptidyl-tRNA hydrolase PTH2
PF01982	CTP-dep_RFKase	Domain of unknown function DUF120
PF01994	Trm56	tRNA ribose 2'-O-methyltransferase, aTrm56
PF02996	Prefoldin	Prefoldin subunit
PF03874	RNA_pol_Rpb4	RNA polymerase Rpb4
PF04019	DUF359	Protein of unknown function (DUF359)
PF04104	DNA_primase_lrg	Eukaryotic and archaeal DNA primase, large subunit
PF04919	DUF655	Protein of unknown function (DUF655)
PF05221	AdoHcyase	S-adenosyl-L-homocysteine hydrolase
PF06026	Rib_5-P_isom_A	Ribose 5-phosphate isomerase A (phosphoriboisomerase A)
PF13393	tRNA-synt_His	Histidyl-tRNA synthetase
PF13656	RNA_pol_L_2	RNA polymerase Rpb3/Rpb11 dimerization domain
PF16906	Ribosomal_L26	Ribosomal proteins L26 eukaryotic, L24P archaeal
PF17144	Ribosomal_L5e	Ribosomal large subunit proteins 60S L5, and 50S L18

Table S3. BC specific cutoff's for pangenome delineation

Bathyarchaeota Clade	GM of Completeness	Minimum Completeness	# MAGs	Relaxed-Core	Shell	Cloud	Singleton
**BC1**	84.01	50.81	41	[41-34]	[33-20]	[19-1]	1
**BC3**	74.17	50.09	12	[12-9]	[8-6]	[5-2]	1
**BC15**	98.13	98.13	1	NA	NA	NA	1
**BC36**	77.91	64.25	17	[17-13]	[12-10]	[9-2]	1
**BC38**	79.69	57.7	12	[12-9]	[8-6]	[5-2]	1
**BC39**	67.32	50.09	3	2	1	NA	1
**BC40**	79.24	67.1	4	3	1	NA	1
**BC41**	81.06	66.83	2	2	1	NA	1
**BC42**	51.29	51.29	1	NA	NA	NA	1

*GM – Geometric Mean

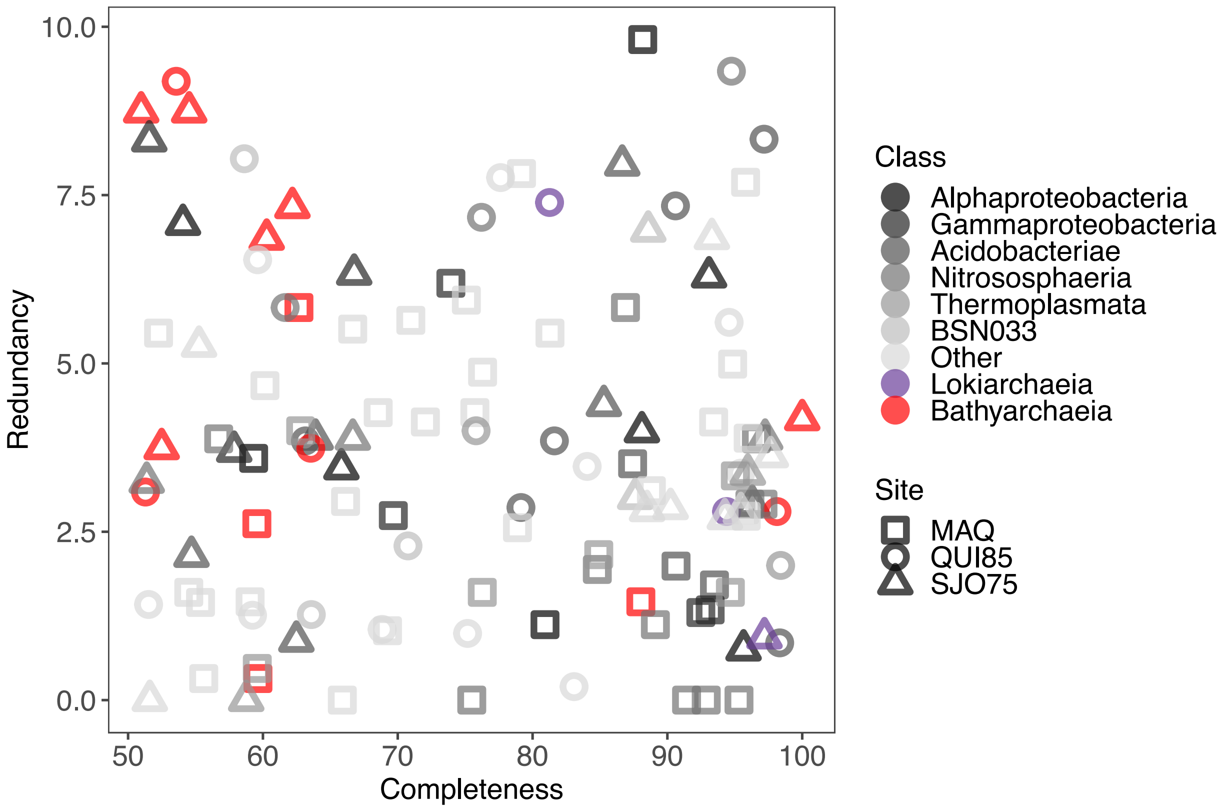

Figure S1. Distribution of HQ and MQ MAGs recovered from the soil metagenomes in this study. Each point represents a different MAG. MAQ – Maquia, QUI85 – Quistococha metagenome from soil core at 85 cm, SJO75 – San Jorge metagenome from solid core at 75 cm.

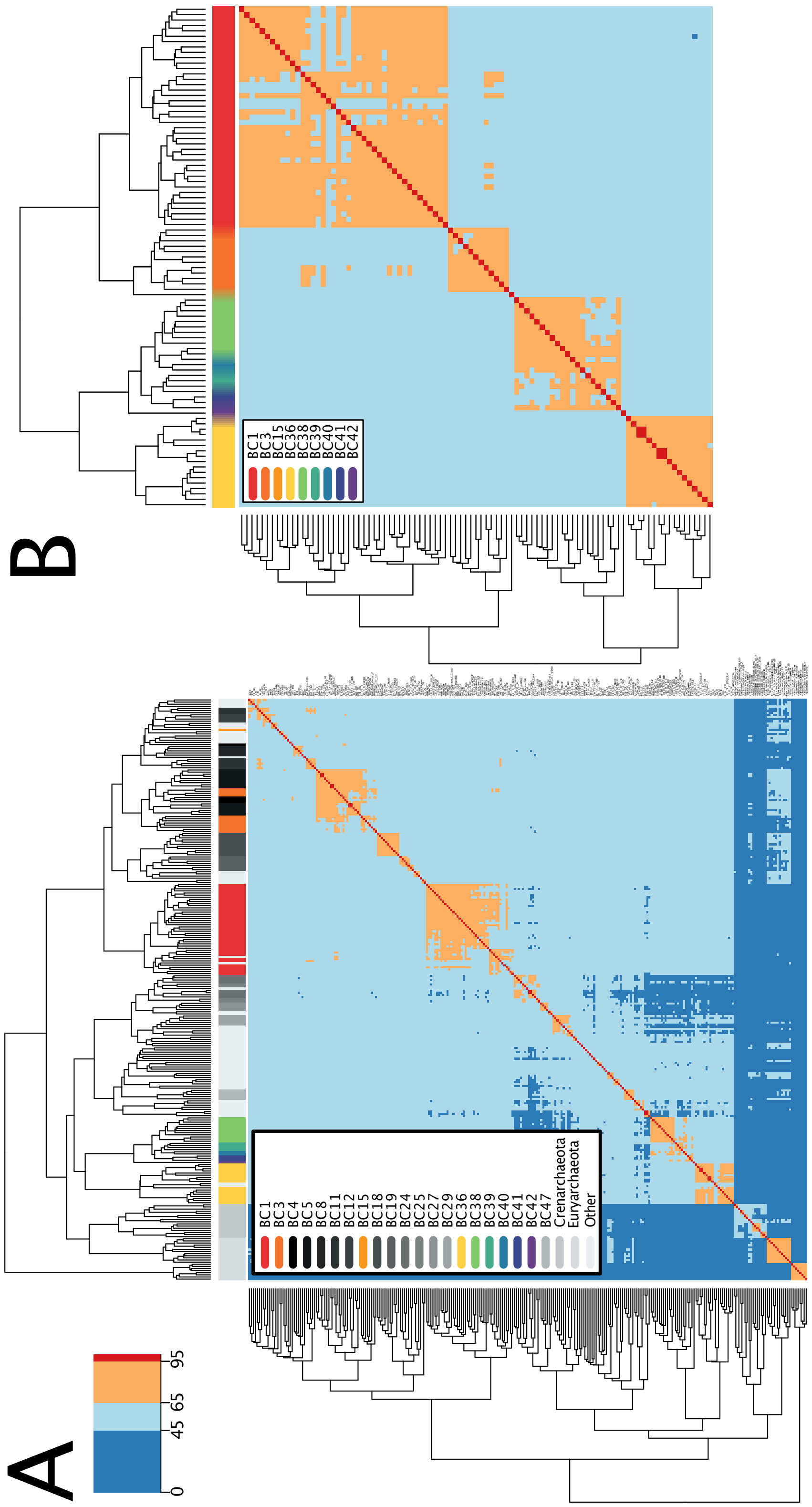

Figure S2. (A) Heatmap showing the percent amino acid identity (AAI) for pairwise MAG comparison for all Bathyarchaeia and out groups in this study. (B) Heatmap of AAI for the nine BC with MAGs from the PMFB. The discrete color heat ramp follows the guidelines outlined in Konstantinidis et al. (2017) for species, genus, and family cutoffs.

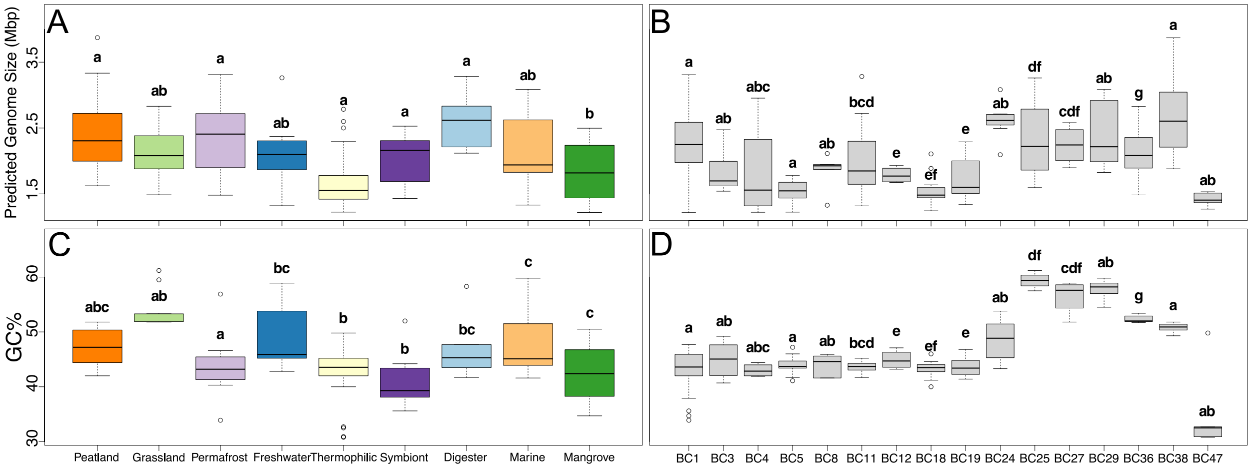

Figure S3. Boxplot of (A) predicted genomes size (Mbp) and (C) GC% for BCs with more than four representative MAGs grouped by recovery ecosystem. Boxplot of the (B) predicted genomes size (Mbp) and (D) GC% for BCs with more than four representative MAGs. Predicted genome size is predicted based on the completeness of MAG. Common letters above boxplot represented non-significant means between BCs calculated by Tukey's multiple comparison at 95% confidence level.

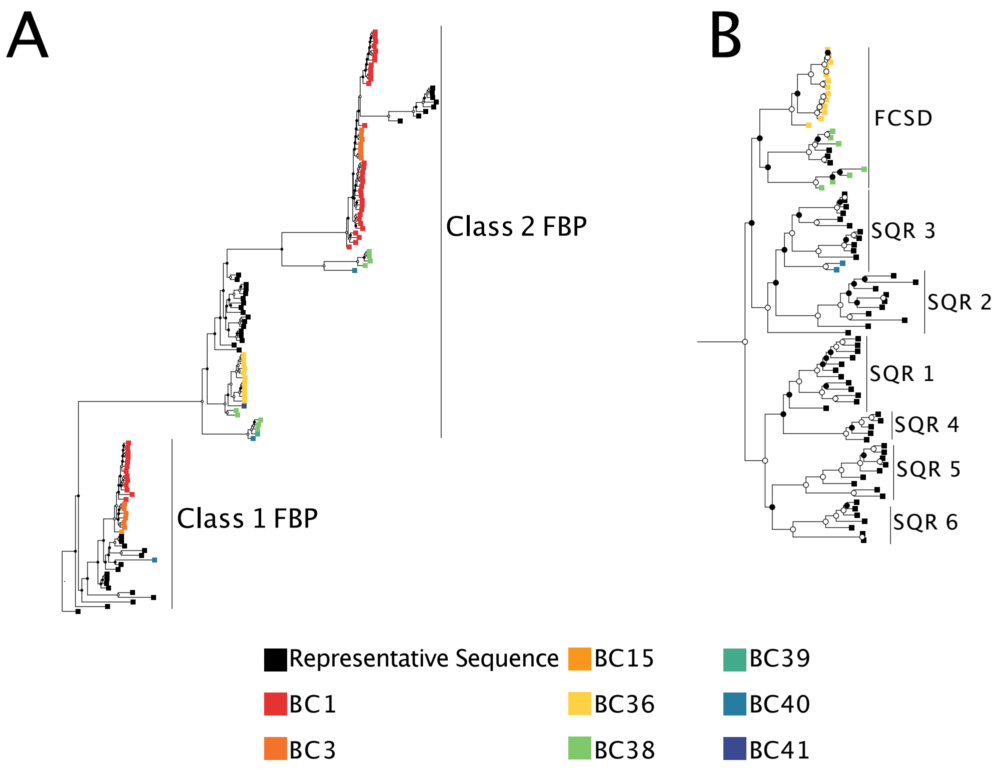

Figure S4. Maximum likelihood inference of fructose bisphosphate (A) and Sulfide:quinone oxidoreductase (B) across Bathyarchaeia MAGs. Robustness was assessed using 300 bootstrapping support. Circles at each node represent bootstrap support of ≥70% (hollow) or <70% (black). Tips are colored according to which clade the sequence was recovered from.

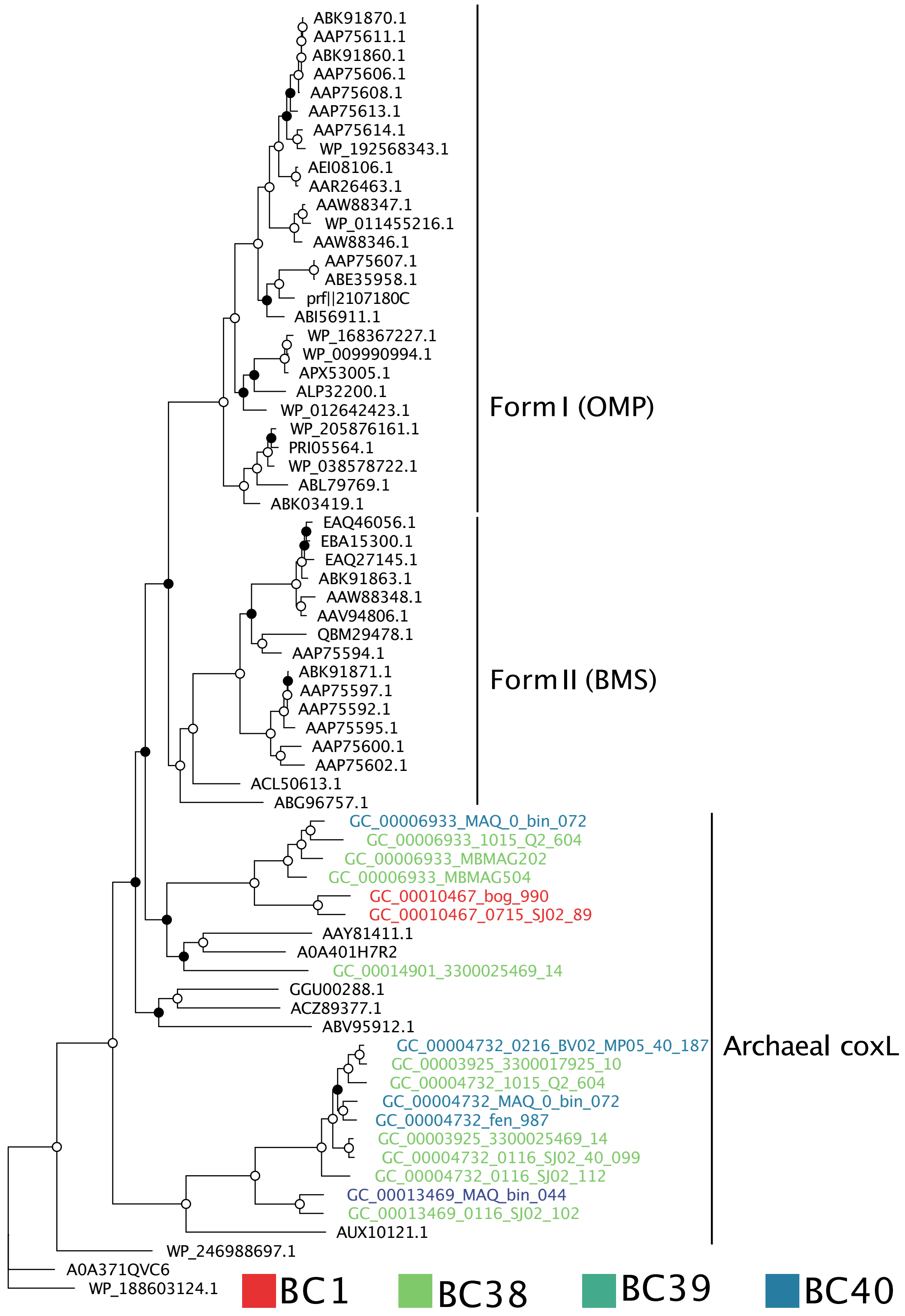

Figure S5. Maximum likelihood inference of CO dehydrogenase large subunit (coxL) recovered from PMFB Bathyarchaeia MAGs. Robustness was assessed using 300 bootstrapping support. Circles at each node represent bootstrap support of ≥70% (hollow) or <70% (black). Tip labels are colored according to which clade the sequence was recovered from. GC_XXXXXXXX before MAG name corresponds to the pangenomic gene cluster.

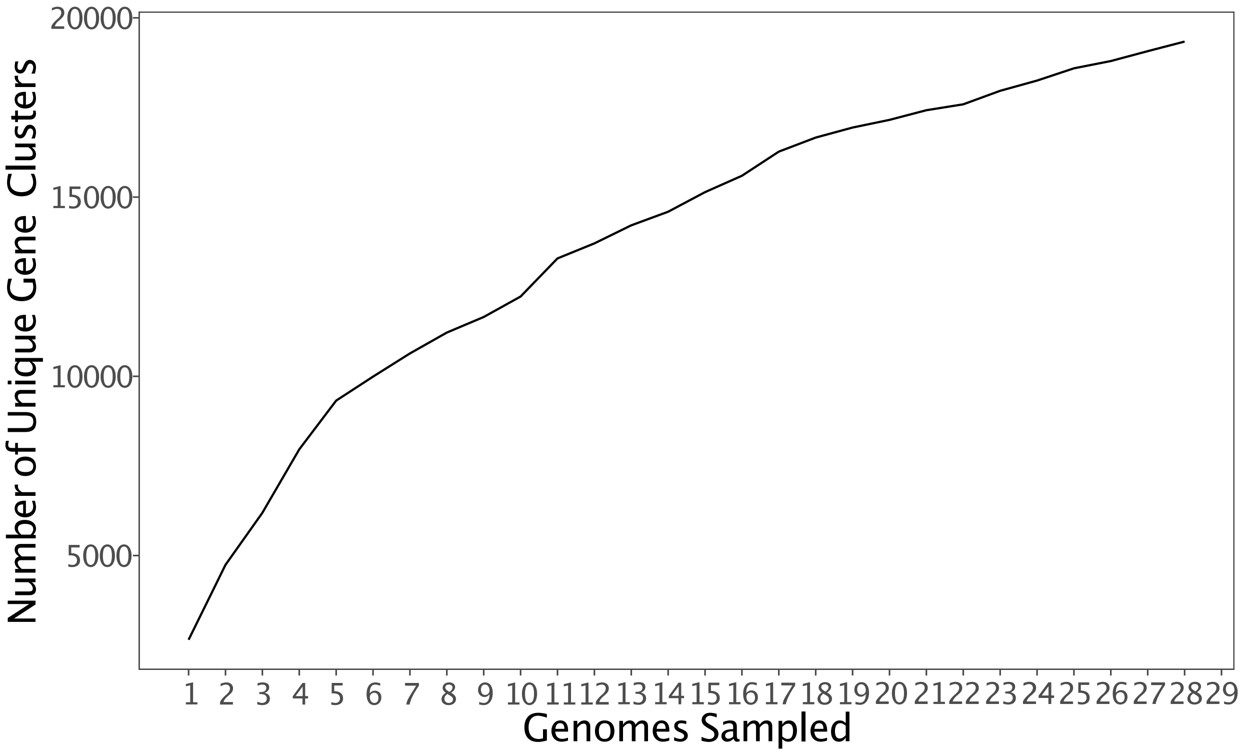

Figure S6. Metapangenome curve of PMFB Bathyarchaeia MAGs.
